# Maternal Genome-Wide DNA Methylation Patterns and Congenital Heart Defects

**DOI:** 10.1371/journal.pone.0016506

**Published:** 2011-01-24

**Authors:** Shimul Chowdhury, Stephen W. Erickson, Stewart L. MacLeod, Mario A. Cleves, Ping Hu, Mohammad A. Karim, Charlotte A. Hobbs

**Affiliations:** Department of Pediatrics, College of Medicine, University of Arkansas for Medical Sciences, Arkansas Children's Hospital Research Institute, Little Rock, Arkansas, United States of America; University of Frankfurt - University Hospital Frankfurt, Germany

## Abstract

The majority of congenital heart defects (CHDs) are thought to result from the interaction between multiple genetic, epigenetic, environmental, and lifestyle factors. Epigenetic mechanisms are attractive targets in the study of complex diseases because they may be altered by environmental factors and dietary interventions. We conducted a population based, case-control study of genome-wide maternal DNA methylation to determine if alterations in gene-specific methylation were associated with CHDs. Using the Illumina Infinium Human Methylation27 BeadChip, we assessed maternal gene-specific methylation in over 27,000 CpG sites from DNA isolated from peripheral blood lymphocytes. Our study sample included 180 mothers with non-syndromic CHD-affected pregnancies (cases) and 187 mothers with unaffected pregnancies (controls). Using a multi-factorial statistical model, we observed differential methylation between cases and controls at multiple CpG sites, although no CpG site reached the most stringent level of genome-wide statistical significance. The majority of differentially methylated CpG sites were hypermethylated in cases and located within CpG islands. Gene Set Enrichment Analysis (GSEA) revealed that the genes of interest were enriched in multiple biological processes involved in fetal development. Associations with canonical pathways previously shown to be involved in fetal organogenesis were also observed. We present preliminary evidence that alterations in maternal DNA methylation may be associated with CHDs. Our results suggest that further studies involving maternal epigenetic patterns and CHDs are warranted. Multiple candidate processes and pathways for future study have been identified.

## Introduction

Birth defects are the leading cause of infant mortality, and congenital heart defects (CHDs) are among the most fatal of all birth defects [1]. Multiple genes have been implicated in CHD development [2], but for the majority of infants diagnosed with a CHD, an established causative gene or teratogenic agent cannot be identified [3], [4]. Identification of risk factors for CHDs are further complicated by the fact that both the maternal and fetal genetic susceptibilities may affect the intrauterine environment during gestation, and both may contribute to the development of CHDs [5], [6]. The complex nature of non-syndromic altered cardiogenesis presents a significant challenge to investigators interested in deciphering the etiology of CHDs.

Maternal folate supplementation has been shown to reduce the risk of CHDs [7]. Folate plays a key role in multiple cellular processes that are in increased demand during pregnancy, including amino acid and nucleotide synthesis, as well as DNA methylation [8]. Previous studies suggest that alterations in DNA methylation may contribute to the development of birth defects [9], [10], [11]. Multiple factors that have been shown to affect DNA methylation patterns including diet, genotype, and environmental exposures [10], [12], [13] have also been recognized as maternal factors implicated in the modification of fetal phenotypes [14], [15], [16]. Maternal genes and environmental exposures may modify the fetus through direct interactions or through alterations in the intrauterine environment during development [17], [18]. Although alterations in maternal one-carbon plasma metabolites indicative of a cellular hypomethylation status have been associated with an increased risk of CHDs [19], [20], a comprehensive study assessing maternal genomic methylation patterns has not yet been conducted.

To understand the mechanisms whereby gene-environment interactions lead to complex disease, epigenetic mechanisms must be considered [21]. DNA methylation is the best characterized epigenetic mechanism. It involves the covalent addition of a methyl group to the cytosine base within the context of CpG dinucleotides. CpG methylation is involved in gene silencing, genomic imprinting, chromosomal stability and protection against parasitic repetitive elements in various cells and tissues [22], [23]. Tightly controlled DNA methylation is essential in early fetal development, and in regulating genomic programming [24], [25]. DNA methylation patterns are tissue-specific and vary depending on cell type [26]. However, DNA methylation patterns of DNA isolated from peripheral blood cells have been shown to be a potential marker of exposure and disease [14], [27].

Through the use of genome-wide DNA methylation array technology, we sought to determine if alterations in maternal gene-specific DNA methylation were associated with CHDs. We then further assessed candidate genes that were differentially methylated between mothers who had CHD-affected pregnancies and control mothers, and identified potential biological processes and pathways of biological relevance to CHDs.

## Results

Genome-wide gene-specific DNA methylation was measured in 367 participants (180 cases and 187 controls). Various lifestyle characteristics were analyzed in our sample population to determine if these differed between cases and controls (**Table 1**). The majority of cases (61.7%) and controls (60.4%) were less than 30 years old. The study population consisted mostly of Caucasian women. Smoking was the only selected covariate that was significantly more prevalent in cases (30.0%) than in controls (18.7%; P = 0.015).

**Table 1:** Selected characteristics of cases and controls.

	Cases (n = 180) n (%)	Controls (n = 187) n (%)	p-value^1^
**Age (y)**			
<30	111 (61.7)	113 (60.4)	
≥30	69 (38.3)	74 (39.6)	0.831
**Race**			
Caucasian	136 (75.6)	148 (79.1)	
African American	32 (17.8)	22 (11.8)	
Others	12 (6.7)	17 (9.1)	0.205
**Smoker**			
Missing	0 (0.0)	1 (0.5)	
No	126 (70.0)	151 (80.7)	
Yes	54 (30.0)	35 (18.7)	0.015
**Alcohol drinker**			
Missing	1 (0.6)	2 (1.1)	
No	93 (51.7)	85 (45.4)	
Yes	86 (47.8)	100 (53.5)	0.294
**Vitamin supplementation**			
Missing	0 (0.0)	1 (0.5)	
No	99 (55.0)	111 (59.4)	
Yes	81 (45.0)	75 (40.1)	0.400
**Maternal education**			
Missing	5 (2.8)	0 (0.0)	
High school or lower	83 (46.1)	79 (42.2)	
College education or higher	92 (51.1)	108 (57.8)	0.342
**Household income**			
Missing	13 (7.2)	12 (6.4)	
Less than $10,000	34 (18.9)	23 (12.3)	
$10,000–$30,000	60 (33.3)	55 (29.4)	
$30,000–$50,000	39 (21.7)	51 (27.3)	
More than $50,000	34 (18.9)	46 (24.6)	0.137
**BMI (kg/m^2^)**			
Missing	8 (4.4)	9 (4.8)	
Underweight (<18.5)	3 (1.7)	6 (3.2)	
Normal (18.5–24.9)	57 (31.7)	78 (41.7)	
Overweight (25.0–29.9)	44 (24.4)	34 (18.2)	
Obese (≥30.0)	68 (37.8)	60 (32.1)	0.118

1 Fisher's exact test for categorical variables.

**Figure 1:**
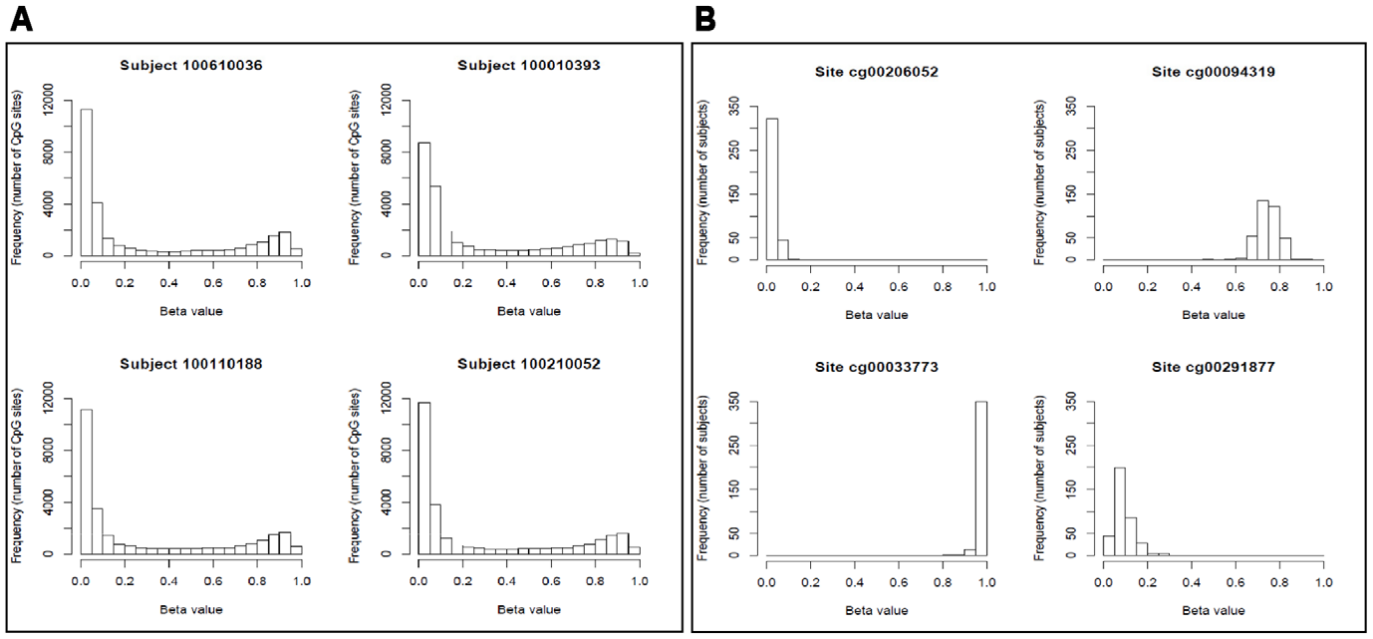
Distribution of β-values for selected subjects and CpG sites. A) The distribution of β values for 27,249 CpG sites is displayed for four randomly selected subjects. The distribution for the four subjects is representative of the distribution for the entire sample population. B) The distribution of β values at selected CpG sites across all 367 study subjects. The four CpG sites were selected to illustrate typical methylation patterns observed in our sample population. Each CpG site displays skewed distributions, with a small amount of variation between individuals. Three of the four panels show substantially skewed distributions, most dramatically for the sites massed nearest 0 or 1. We did not observe drastic shifts in DNA methylation patterns in our sample population at individual CpG sites.

To illustrate the representative distribution of methylation levels across more than 27,000 CpG sites, **Figure 1A** displays histograms of β-values for four randomly selected study subjects. In each sample, the distribution is bimodal with a high peak of hypomethylated CpG sites and a low peak of hypermethylated CpG sites. These individuals, therefore, have a large proportion of CpG sites characterized by low methylation, a smaller proportion of sites characterized by high methylation, and a certain proportion of sites in between the two peaks with moderate levels of methylation. The distributions displayed are typical of all subjects. The observed distribution of β-values was expected as the array design is biased to interrogate the promoter regions of genes within CpG islands.

**Figure 1B** displays histograms of β-values at selected CpG sites across all study subjects. The four CpG sites were selected to illustrate typical methylation patterns observed in our sample population. Each CpG site has a typical methylation level, with a small amount of variation between individuals. Three of the four panels in **Figure 1B** show substantially skewed distributions, most dramatically for the data massed nearest 0 or 1. We did not observe drastic shifts in DNA methylation patterns in our sample population at individual CpG sites.

To determine if the associations between DNA methylation and cardiac defects were related to specific cardiac phenotypes, an analysis stratified by cardiac phenotypes was conducted (data not shown). Cardiac phenotypes included conotruncal, septal, and obstructive defects. Cases that had more than one cardiac defect type were included in all relevant strata. Among cases, 47.2% had atrial or ventricular septal defects (n = 85), 35.4% had right- and left-sided obstructive defects (n = 63), and 16.7% had conotruncal defects (n = 30). The 14 cases that did not fall within one of the three described main defect groups were excluded from the stratified analysis due to a small sample size. Analyses with sub-phenotype indicator variables included in the regression model were conducted and no statistically significant findings of sub-phenotype-specific methylation patterns were observed. Because gene-specific methylation patterns did not deviate significantly between cardiac phenotypes, cases were combined for subsequent analyses to maximize power to test our study hypotheses.

**Figure 2:**
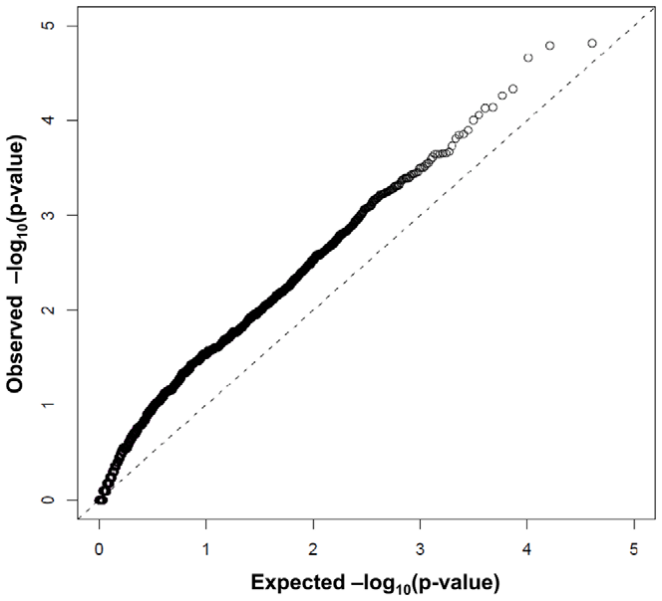
Quantile-Quantile (Q-Q) plot of observed versus expected p-values. A quantile-quantile (Q-Q) plot of –log_10_(p-values) for the tests of association between gene-specific methylation and disease status. The observed quantiles are consistently higher than their expected values under the null hypothesis of no disease association, providing evidence of site-specific disease association for a large number of CpG sites.

**Figure 2** displays a quantile-quantile (Q-Q) plot of –log_10_(p-values) for the 27,249 tests of association between gene-specific methylation and disease status. The observed quantiles are consistently higher than their expected values under the null hypothesis of no disease association, providing evidence of site-specific disease association for a large number of CpG sites.

We identified 425 CpG sites that were differentially methylated between cases and controls with P<0.005, although an FDR threshold of 0.05 was not reached for any single CpG site (**Table S1**). The top 50 CpG sites ranked by statistical significance are listed in **Table 2**. The 425 CpG sites of interest encompassed 415 genes. The vast majority of these CpG sites were located within CpG islands (386 CpG sites, 90.8%). Given that the array platform contains 76% of sites within CpG islands, this result was statistically significant (P<0.001). Additionally, we observed that case mothers were hypermethylated in the majority of loci (379 sites, 89.2%) compared to hypomethylated loci (46 sites, 10.8%). Finally, the list of differentially methylated genes includes four genes previous shown to be imprinted (**Table 3**) as well as 14 miRNA sites (**Table 4**).

**Table 2:** Top 50 differentially methylated genes ranked by statistical significance.

SYMBOL	Gene name	Gene_ID	p-value^1^	q-value^2^	Chr^3^	CpG Island	Direction^4^
CTSL2	cathepsin L2	1515	1.6E-05	0.13378	9	Yes	+
RREB1	ras responsive element binding protein 1	6239	1.6E-05	0.13378	6	Yes	+
BSN	bassoon (presynaptic cytomatrix protein)	8927	2.2E-05	0.13378	3	Yes	+
HSPC268	hypothetical protein HSPC268	154791	4.6E-05	0.17086	7	Yes	+
NEUROG1	neurogenin 1	4762	5.5E-05	0.17086	5	Yes	+
MAP4K5	mitogen-activated protein kinase kinase kinase kinase 5	11183	7.3E-05	0.17086	14	Yes	+
FTMT	ferritin mitochondrial	94033	7.4E-05	0.17086	5	Yes	-
CPE	carboxypeptidase E	1363	8.8E-05	0.17086	4	Yes	+
EXT2	exostosin 2	2132	0.0001	0.17086	11	Yes	+
UBE3A	ubiquitin protein ligase E3A	7337	0.00013	0.17086	15	Yes	+
DNAJB6	DnaJ (Hsp40) homolog, subfamily B, member 6	10049	0.00014	0.17086	7	Yes	+
UQCRH	ubiquinol-cytochrome c reductase hinge protein	7388	0.00014	0.17086	1	Yes	+
ORF1-FL49	chromosome 5 open reading frame 32	84418	0.00016	0.17086	5	Yes	+
H2AFY2	H2A histone family, member Y2	55506	0.00018	0.17086	10	Yes	+
PKN2	protein kinase N2	5586	0.00022	0.17086	1	Yes	+
STRN3	striatin, calmodulin binding protein 3	29966	0.00022	0.17086	14	Yes	+
C8orf35	chromosome 8 open reading frame 35	55174	0.00022	0.17086	8	Yes	+
NDUFA7	NADH dehydrogenase (ubiquinone) 1 alpha subcomplex, 7	4701	0.00023	0.17086	19	Yes	-
SLC36A4	solute carrier family 36 (proton/amino acid symporter), member 4	120103	0.00023	0.17086	11	Yes	+
C6orf72	chromosome 6 open reading frame 72	116254	0.00023	0.17086	6	Yes	+
C6orf199	chromosome 6 open reading frame 199	221264	0.00025	0.17086	6	Yes	+
CCDC74A	coiled-coil domain containing 74A	90557	0.00026	0.17086	2	Yes	+
TERF1	telomeric repeat binding factor (NIMA-interacting) 1	7013	0.00028	0.17086	8	Yes	+
SNX3	sorting nexin 3	8724	0.00028	0.17086	6	Yes	+
HNRPH3	heterogeneous nuclear ribonucleoprotein H3 (2H9)	3189	0.0003	0.17086	10	Yes	+
CGNL1	cingulin-like 1	84952	0.00031	0.17086	15	Yes	-
ITM2B	integral membrane protein 2B	9445	0.00031	0.17086	13	Yes	+
ZNF544	zinc finger protein 544	27300	0.00032	0.17086	19	Yes	+
MGC14376	hypothetical protein MGC14376	84981	0.00035	0.17086	17	Yes	+
ICAM3	intercellular adhesion molecule 3	3385	0.00036	0.17086	19	No	+
RRP22	Ras-related protein 22	10633	0.00036	0.17086	22	Yes	+
AGTPBP1	ATP/GTP binding protein 1	23287	0.00037	0.17086	9	Yes	+
WNT5A	wingless-type MMTV integration site family, member 5A	7474	0.00037	0.17086	3	Yes	+
GMDS	GDP-mannose 4,6-dehydratase	2762	0.00039	0.17086	6	Yes	+
XPR1	xenotropic and polytropic retrovirus receptor 1	9213	0.0004	0.17086	1	Yes	+
GDF3	growth differentiation factor 3	9573	0.00041	0.17086	12	Yes	+
RASEF	RAS and EF-hand domain containing	158158	0.00041	0.17086	9	Yes	+
KCTD4	potassium channel tetramerisation domain containing 4	386618	0.00041	0.17086	13	No	-
RFXAP	regulatory factor X-associated protein	5994	0.00041	0.17086	13	Yes	+
KCNA4	potassium voltage-gated channel, shaker-related subfamily, member 4	3739	0.00042	0.17086	11	Yes	+
EGFR	epidermal growth factor receptor	1956	0.00043	0.17086	7	Yes	+
LGR6	leucine-rich repeat-containing G protein-coupled receptor 6	59352	0.00045	0.17086	1	Yes	+
MAPK13	mitogen-activated protein kinase 13	5603	0.00047	0.17086	6	Yes	+
SSFA2	sperm specific antigen 2	6744	0.00049	0.17086	2	Yes	+
PHCA	phytoceramidase, alkaline	55331	0.00049	0.17086	11	Yes	+
TBC1D1	TBC1 (tre-2/USP6, BUB2, cdc16) domain family, member 1	23216	0.00049	0.17086	4	Yes	+
LRRC3B	leucine rich repeat containing 3B	116135	0.00049	0.17086	3	Yes	+
SHCBP1	SHC SH2-domain binding protein 1	79801	0.0005	0.17086	16	Yes	+
PMS2	PMS2 postmeiotic segregation increased 2 (S. cerevisiae)	5395	0.00053	0.17086	7	Yes	+
ZNF304	zinc finger protein 304	57343	0.00053	0.17086	19	Yes	+

1 p-values were calculated by multiple linear regression and randomization testing (see Materials and Methods).

2 q-values were calculated using the *qvalue* package in the R statistical programming environment under default settings (see Materials and Methods).

3 Chr =  chromosome.

4 Direction of methylation in cases.

**Table 3:** Differentially methylated imprinted genes.

SYMBOL	Gene function	p-value^1^	q-value^2^	Direction of methylation in cases	Chromosome	CpG Island
**UBE3A**	Imprinted	0.0001262	0.1708606	+	15	Yes
**ZNF264**	Imprinted	0.0008294	0.1743975	+	19	Yes
**SNURF**	Imprinted	0.0039526	0.1822456	-	15	Yes
**INS**	Imprinted	0.0043908	0.1822456	-	11	No

1 p-values were calculated by multiple linear regression and randomization testing (see Materials and Methods).

2 q-values were calculated using the *qvalue* package in the R statistical programming environment under default settings (see Materials and Methods).

**Table 4:** Differentially methylated miRNAs.

Gene	miRNA	p-value^1^	q-value^2^	Direction of methylation in cases	Chromosome	CpG Island
**HOXB4**	HSA-MIR-10A;	0.0043384	0.1822456	+	17	Yes
**BTG4**	HSA-MIR-34B;HSA-MIR-34C;	0.0006663	0.1708606	+	11	Yes
**CCDC55**	HSA-MIR-423;	0.0029542	0.1822456	-	17	Yes
**SACM1L**	HSA-MIR-565;	0.0008043	0.1743975	+	3	Yes
**HSPC268**	HSA-MIR-594;	0.0000463	0.1708606	+	7	Yes
**HSPC268**	HSA-MIR-594;	0.0045767	0.1822456	+	7	Yes
**WDR37**	HS_104;	0.0033306	0.1822456	+	10	Yes
**DUSP4**	HS_128;	0.0034043	0.1822456	+	8	Yes
**C10orf4**	HS_138;	0.0026144	0.1822456	+	10	Yes
**PIGO**	HS_140;	0.0044543	0.1822456	+	9	Yes
**PPME1**	HS_176;	0.0015582	0.1822456	+	11	Yes
**DUS2L**	HS_253;HS_253;	0.0041322	0.1822456	-	16	Yes
**CDC7**	HS_255;	0.0049322	0.1822456	+	1	Yes
**FBXO28**	HS_78;	0.0015055	0.1822456	+	1	Yes

1 p-values were calculated by multiple linear regression model and randomization testing (see Materials and Methods).

2 q-values were calculated using the *qvalue* package in the R statistical programming environment under default settings (see Materials and Methods).

Further analysis of the differentially methylated genes was conducted for potential biological significance. Gene Set Enrichment Analysis (GSEA) was performed to determine if the differentially methylated genes were significantly enriched in relevant biological processes and pathways of biological relevance to fetal heart development.

**Figure 3:**
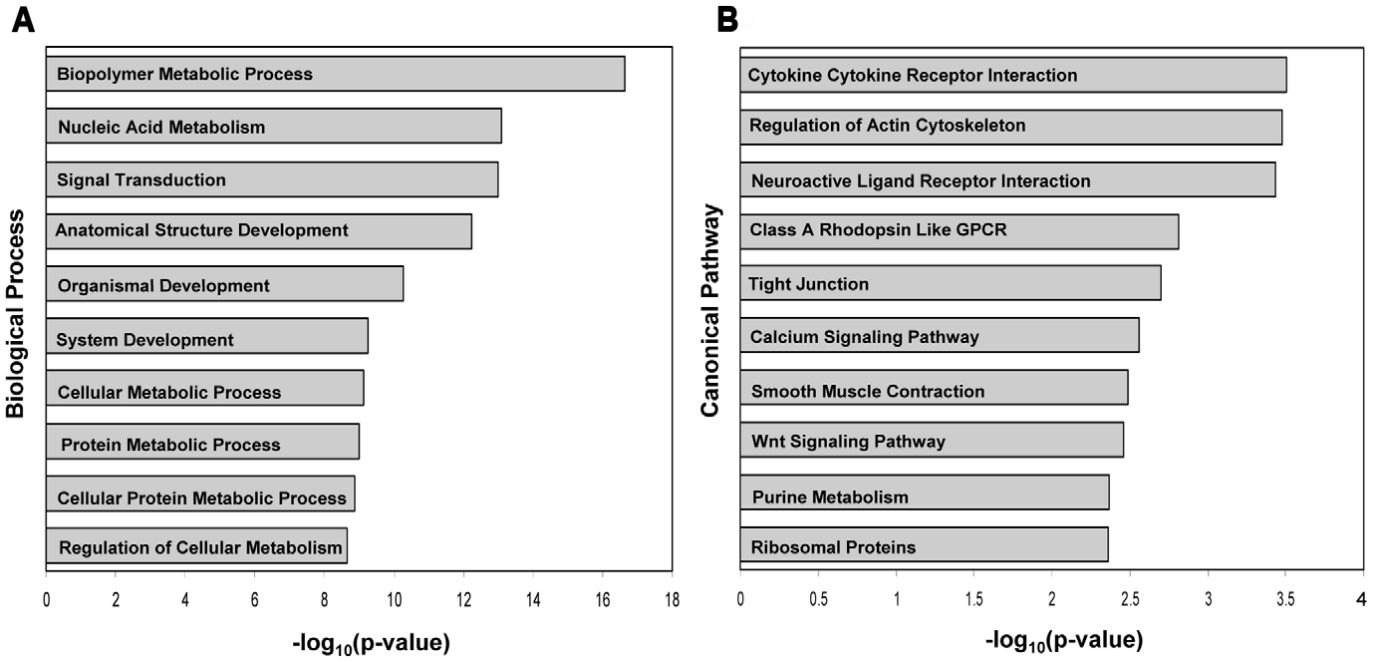
Gene Set Enrichment Analysis (GSEA) of biological processes and canonical pathways for differentially methylated genes. A) The top ten significantly enriched biological processes are displayed with their corresponding –log_10_(p-value). Multiple processes appear to be directly related to fetal heart development. B) The top ten significantly enriched canonical pathways are displayed with the corresponding −log_10_(p-value). Multiple pathways had previously been implicated in CHDs.

The 415 genes of interest were input into the GSEA program, and the top ten gene set overlaps were generated. **Figure 3A** summarizes the results of the most significant overlaps for the biological process analysis. Within the top ten most significant gene set overlaps, multiple functional categories associated with development were strongly enriched within our differentially methylated gene list. Specifically, the biological process categories of nucleic acid metabolism, signal transduction, anatomical structure development, multicellular organismal development, and system development have potential direct functional implications in the development of CHDs. The inclusion of these specific processes within the top ten most significant gene set overlaps provided evidence of strong biological relevance of the differentially methylated genes to fetal development. GSEA was also conducted to determine if significant canonical pathway overlaps could be established from the differentially methylated gene list (**Figure 3B**). Pathway analysis revealed functional overlaps that have previously been shown to be involved in embryonic heart development. These pathways included cytokine-cytokine receptor interaction, the regulation of actin cytoskeleton, tight junction, calcium signaling pathway, smooth muscle contraction and the *Wnt* signaling pathway. The GSEA results provided further evidence that the genes that were found to be differentially methylated appear to have biological relevance to CHDs. The results summaries generated from the GSEA, which includes the specific differentially methylated genes involved within each biological process and canonical pathway overlap, is included in **Table S2** and **Table S3** respectively.

## Discussion

To our knowledge, we have performed the first population based maternal case-control study to provide evidence of association between maternal gene-specific DNA methylation and CHDs. Further analysis of the differentially methylated genes revealed functionally relevant enrichment in biological processes and pathways previously shown to be involved in fetal development.

No individual CpG site achieved genome-wide statistical significance. A combination of low variation in site-specific methylation between individuals and relatively modest sample size may account for the inability to reach statistical genome-wide significance. Thus, caution in interpreting the results of differentially methylated genes of interest must be noted. The advent of genome-wide association studies (GWAS) has led to an influx of studies structured to detect genetic variants associated with various human diseases, but limited studies have been conducted using genome-wide DNA methylation platforms. Issues regarding adjustments for multiple testing, statistically significant thresholds, and biologically relevant alteration levels, have not been established for high-throughput DNA methylation studies. The increased demand for genome wide epigenetic studies will lead to clearer definition of these issues in the future.

In the current study, gene-specific methylation was measured in DNA isolated from peripheral blood lymphocytes. We observed slight alterations in methylation between cases and controls. Factors such as genetic variation, metabolites involved in one-carbon metabolism, and environmental exposures are possible mechanisms of differential methylation between cases and controls. The interpretation of results from DNA methylation studies must always be evaluated with caution as epigenetic marks may vary depending on cell type and can be influenced by various factors. Previous studies have used Illumina Infinium technology to assess DNA methylation in peripheral blood lymphocytes and have identified CpG sites associated with disease [27], [28]. The accumulation of studies that have assessed DNA methylation patterns in lymphocytes in multiple diseases and exposures provides evidence that methylation patterns in peripheral blood cells may be useful in identifying biomarkers of various diseases.

It has been established that various maternal factors may cause altered fetal growth and development [29], but the impact of maternal genetics and epigenetics in the development of birth defects is not well understood. Maternal genes may exert effects through direct interactions with the fetus [30], [31] or through changes to the intrauterine environment. Maternal blood provides nutrients to the developing fetus and could provide an avenue of signal exchange. Multiple studies have been conducted to determine the impact of maternal genetic effects and the risk of birth defects [32], [33], [34]. Although these studies encompass a relatively small number of genes with respect to the genome, they suggest that maternal genetic effects, independent of fetal genotype, may influence the risk of birth defects. Additionally, alterations in gene expression in maternal blood are associated with adverse pregnancy outcomes [35], [36] providing further evidence that maternal genetic influence may impact the fetus. Epigenetics are considered to be the link between environmental exposures and their effects on the genome [37] and may explain how maternal genes may exert effects on the developing fetus.

Genes previously implicated in CHDs were identified within our differentially methylated genes. For example, multiple genes involved in the mitogen-activated protein kinases (MAPK) pathway are included in the list of differentially methylated genes. The MAPK pathway is involved in multiple cellular processes including transcription, proliferation, migration, survival, and differentiation [38]. It has been postulated that this family of genes may play a role in CHDs [39]. Additional genes implicated in heart development that were differentially methylated in our study include, but are not exclusive to, *EGFR* [40], *GATA4* [41], and *Wnt5a* [42]. The inclusion of multiple miRNA targets as candidate genes is intriguing as the diverse role of miRNAs in cardiac function has received increased interest [43]. Although genetic studies have been conducted on these genes of interest, our results indicate that epigenetics should be considered as well.

Alterations in maternal DNA methylation may also indirectly affect the fetus through changes in the maternal environment. Changes in the maternal environment can result in direct changes in gene expression in the developing fetus [44], and if certain harmful exposures occur during organogenesis, various structural anomalies may occur [13]. Alterations in maternal methylation in genes involved in the maintenance of the intrauterine environment may lead to an increased susceptibility to teratogenic agents. Genes that were found to be differentially methylated, such as *GPX3*, provide an example of a gene involved in the sustainment of a normal maternal environment. The *GPX3* gene is involved in antioxidant potential in the body. Altered expression in *GPX3* may result in a lower anti-oxidant potential, which may potentially lead to increased oxidative stress for the fetus.

It is though that hypomethylation in the parental germline can influence genomic instability, and that this instability may subsequently cause genomic instability in the progeny [45]. Although it is unclear how maternal lymphocyte methylation patterns correlate with germ cells or the fertilized oocyte, maternal methylation patterns in peripheral blood DNA have recently been shown to correlate with infant methylation patterns in certain loci [46], [47]. The phenomenon of passing intact methylation patterns to future generations, termed transgenerational epigenetic inheritance, has been used to explain non-Mendelian inheritance patterns in complex diseases. Although speculation regarding whether epigenetic marks are transmitted intact from parent to offspring exists [48], the vast majority of the evidence is found in animal models [49].

The importance of epigenetic factors in the development of complex disease is now unquestioned. Embryonic heart development requires a series of highly complex, coordinated and rapid morphogenesis processes. Specifically for CHDs, epigenetic factors may contribute to cardiomyocyte differentiation and chamber morphogenesis [50], and it has been proposed that epigenetic mechanisms are necessary to coordinate genetic programs in heart development [51]. The complex series of events involved in heart development indicates that multiple genes and pathways are responsible for CHDs [52]. Although many studies have been conducted to investigate the developmental genetics of CHDs, limited studies are available regarding DNA methylation within these genes and pathways. Our analysis of biological processes revealed overlaps involving system, anatomical structure, and organismal development. The listed processes are involved in fetal development and provide candidate processes for additional epigenetic studies. Nucleic acid metabolism and signal transduction are also of importance to development as the increase demand for DNA synthesis and properly functioning signaling cascades are important during pregnancy [8], [53]. Canonical pathways enriched within our genes of interest have previously been implicated in heart development and some of the pathways appear to be interconnected. For example, alterations in intracellular calcium levels can affect smooth muscle contraction downstream [54]. Other significant pathways that have previously been implicated in heart development include ribosomal proteins [55], the actin cytoskeleton [52], *Wnt* signaling [56], and cytokine-cytokine receptor interaction [57]. Future epigenetic studies should be conducted within the candidate pathways and processes described.

Certain methodological limitations of this study should be considered. The blood obtained to isolate genomic DNA was collected after pregnancy. We have previously described the rationale for the use of case-control study designs in human birth defects research studies [20]. We did not measure DNA methylation changes during gestation and neonatal development, which represents a period of high epigenomic susceptibility [58]. Contrasting reports exist regarding gene-specific methylation changes over time [59], [60], and it is unclear how maternal methylation patterns may change during pregnancy. Another limitation of our study is the lack of infant methylation data to determine if differential methylation is observed between infants with CHDs and controls, and if these methylation patterns are associated with maternal DNA methylation patterns. Previous studies have shown global DNA hypomethylation in the proband for neural tube defects [61], [62], but studies in gene-specific and global methylation in a family-based design should be conducted for other congenital anomalies including CHDs. The design of the HumanMethylation27 BeadChip allows the assessment of over 27,000 CpG sites, which represents 0.01–0.18% of the single-copy genome [48]. The assay is focused primarily on one or two CpG sites within gene promoter regions. Thus, most of the neighboring CpG sites are not studied. Although our study has revealed a strong bias in DNA alterations occurring in CpG islands, the possibly exists for DNA methylation alterations occurring outside CpG islands and in CpG sites not included on the assay. Although the Illumina Infinium HumanMethylation27 platform provides a high-throughput and a reliable examination of a large number of genes [63], studies in birth defects investigating a larger number of CpG sites should be conducted. Our study provides additional evidence that maternal epigenetics and genetics should be included in the investigation of birth defects.

Alterations in maternal DNA methylation converge with evidence from previous studies that indicate folate-dependent genetic and metabolic susceptibilities increase the risk of CHDs. The study has notable strengths including analyzing genome-wide gene specific DNA methylation in a large population of mothers. Extensive statistical modeling was used to adjust for any potential experimental confounders. We also adjusted for various lifestyle factors that have been shown to affect DNA methylation. The data presented here strongly suggest that maternal epigenetics may be a determinant of CHD risk. The non-invasive nature of the determination of DNA methylation in lymphocytes isolated from peripheral blood draw makes it an attractive candidate for disease risk assessment. Identification of genetic and epigenetic profiles associated with an increased risk of CHDs may add new dimensions to preconception risk assessment as well as further elucidate mechanisms involved in CHD development. Because DNA methylation may be influenced by diet or drugs, epigenetics may provide an avenue for future therapeutic intervention and prevention of CHDs.

## Materials and Methods

### Study Population

The National Birth Defects Research and Prevention Study (NBDPS) is an ongoing multi-site population-based case-control study investigating the etiology of 30 non-syndromic birth defects [64]. The NBDPS is the largest case-control study of birth defects ever conducted in the US. The study population and eligibility criteria for the NBDPS have been previously outlined [20]. For the current study, case women were Arkansas residents who participated in the NBDPS and who delivered a singleton live birth with a non-syndromic CHD. Cases for whom the pregnancy was also affected by a known single gene disorder, chromosomal abnormality, or syndrome were excluded. All diagnostic tests on cardiac NBDPS case infants were reviewed by a pediatric cardiologist to ensure uniform criteria were used for diagnoses. To be eligible for the study described, case infants were required to have at least one cardiac lesion, which included conotruncal, septal, and/or obstructive lesions. Using a classification system developed for the NBDPS, which incorporated three dimensions of cardiac phenotype, cardiac complexity, and extracardiac anomalies [65], case women for the current study included those who carried fetuses with simple or association CHDs. Control mothers were Arkansas residents who had a singleton live birth without birth defects during the same period as the cases, who participated in the NBDPS, and were randomly selected from birth certificate data or hospital discharge logs. Case and control mothers spoke either English or Spanish.

From eligible Arkansas NBDPS participants, we assessed DNA methylation in 180 cases and 187 controls for whom frozen blood samples were available. Blood samples were collected by research nurses during home visits in Arkansas that occurred between 2001 and 2008. Samples were collected by venipuncture after pregnancy. All participants signed informed consent approved by the UAMS Institutional Review Board.

### Covariates

Information regarding selected lifestyle factors was obtained from in-home interviews conducted by research nurses using a Block Abbreviated Food-Frequency Questionnaire [66] and from a NBDPS-structured computer-assisted telephone interview. The information on covariates reflects data up to one month prior to the visit. Variables considered as covariates in this study due to their potential effects on DNA methylation status were age, race, body mass index (BMI), and the use of multivitamins, cigarettes, and alcohol.

### Sample preparation and processing

Fasting blood samples were collected in EDTA-Vacutainer tubes and immediately chilled on ice before they were centrifuged at 4000×g for 10 min at 4°C. DNA was isolated from the stored frozen blood samples according the manufacturer's protocol for the PureGene DNA isolation kit (*Gentra Systems*, Minneapolis, MN).

### DNA quantification and bisulfite conversion

Genomic DNA was quantified via the RNase P 20x assay (*Applied Biosystems*, Forest City, CA). In a total reaction volume of 25 µl, 2 µl of genomic DNA was used for absolute quantification for the RNase P assay on the ABI 7900HT Real Time PCR System, according to the manufacturer's protocol. After genomic DNA quantification, 500 ng of genomic DNA underwent bisulfite modification utilizing the EZ DNA Methylation-Direct Kit (*Zymo Research*, Orange, CA). The bisulfite-converted DNA was resuspended in 12 µl TE buffer and stored at -80°C until the samples were ready for analysis.

### Infinium Assay for Methylation

DNA methylation analysis was conducted using the Illumina Infinium Human Methylation27 BeadChip. Each HumanMethylation27 BeadChip consists of 12 arrays, and up to 4 bead chips were processed simultaneously. The assay allows the interrogation of over 27,000 CpG sites located within the proximal promoter regions of over 14,000 consensus coding sequences (CCDS) genes throughout the genome [63]. In addition, the assay includes 110 miRNA promoters and also includes imprinted genes.

The standard protocol provided by Illumina was used for DNA methylation analysis. Briefly, 4 µl of bisulfite converted DNA was isothermally amplified at 37°C overnight. The amplified DNA product was fragmented by an endpoint enzymatic process. Fragmented DNA was precipitated, resuspended, and applied to an Infinium Human Methylation27 BeadChip and hybridized overnight. During hybridization, the amplified and fragmented DNA samples anneal to specific oligomers that are covalently linked to over 27,000 different bead types. Each bead type corresponds to the nucleotide identity and thus the methylation status at a bisulfite-converted cytosine in a specific CpG site. The bead chips were then subjected to a single-base extension reaction using the hybridized DNA as a template incorporating fluorescently labeled nucleotides of two different colors, each corresponding to the cytosine (methylated) or uracil (unmethylated) identity of the bisulfite-converted nucleotide at a specific CpG site. The fluorescently stained chip was imaged by the Illumina BeadArray Reader. Illumina's Genome Studio program was used to analyze BeadArray data to assign site-specific DNA methylation β-values to each CpG site. The proportion of methylation (β) for each subject at each CpG site was computed by first subtracting the background signal intensity of negative controls from both the methylated and unmethylated signals and then taking the ratio of the methylated signal intensity to the sum of both methylated and unmethylated signals. Thus, the β-value is a continuous variable ranging between 0 and 1.

### Experimental Design and Quality Control

A total of one batch of two bead chips (24 samples) and eight batches of four bead chips were assayed. Cases and controls were randomly placed within the bead chips. Replicate samples were included in each batch for quality control and normalization measures. All replicate samples achieved a correlation coefficient of greater than 0.985 (data not shown). Using Illumina's Genome Studio, background adjusted β-values and assay control probe information was used for initial quality control. Subjects were required to pass three initial quality control criteria in order to be included in further analysis: 1) Subjects achieved a 95% CpG site call rate, which equals 26,250 sites called at an α<0.05 significance level; 2) Background signal for each subject was under 1000 units, determined from the controls dashboard in Genome Studio; and 3) Clear separation was observed in the non-polymorphic controls, also determined from the controls dashboard. Samples that did not meet these criteria were removed from the analysis. Individual CpG sites were examined using the detection p-value metric provided by Illumina in which the signal generated from each CpG site is compared to negative controls. A threshold of p<0.05 was used as a cutoff. CpG sites that did not reach this threshold were eliminated from the analysis. Using this criterion, 329 of 27,578 CpG sites (1.2%) had missing or invalid β-values in ten or more samples; these sites were dropped from subsequent analysis, leaving 27,249 CpG sites among 367 subjects.

### Principal Component Analysis

Principal component analysis (PCA) was performed on the β-value data matrix of 367 subjects (rows) and 27,249 CpG sites (columns). Data were logit-transformed prior to PCA and subsequent association testing. As described above, 329 CpG sites with missing or invalid β-values in ten or more samples were removed from the data; before performing PCA, any remaining missing β-values were imputed using *k* nearest neighbor averaging [67].

The first 20 principal components (PCs) explain 62.8% of overall variance in the logit-transformed β-value matrix, and no single PC among the remaining 347 PCs explains more than 0.34% (**Figure S1**). Regression analysis (not shown) revealed that these 20 PCs are variously associated with experimental factors that had been previously shown to be potential confounders under the Infinium Methylation platform [27], as well as with several phenotypic and lifestyle factors, including case/control status, age, race, and body mass index (BMI), and the use of multivitamins, cigarettes, and alcohol. Regression analysis of the top 20 PCs therefore informed the selection of covariates to be included in the CpG-site-specific regression model, which is described in the following section.

### Determination of differentially methylated sites between cases and controls

After initial quality control and PCA of the β value data, a sample of 367 subjects (180 cases and 187 controls) was tested for association between disease status (i.e. case/control) and maternal gene-specific methylation. In this analysis, each CpG site was tested for association by regressing its logit-transformed β value on case/control status using multiple linear regression models. Additional covariates included these models were experimental batch (as a factor), bisulfite-conversion (BSC) efficiency, age, BMI, race (as a factor), and indicator variables of perinatal vitamin usage, alcohol drinking, and tobacco smoking. BSC efficiency for each subject has two channels, red and green. The red and green channels are highly correlated but are not sufficiently to be collinear, thus both channels were included in the models (**Figure S2**).

Under standard theory a *t*-statistic, derived from the least-squares estimate of the case/control parameter and its standard error, was compared with the theoretical *t*-distribution to test the null hypothesis of no association between disease status and site-specific methylation, 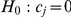, where 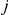 = 1,…,27,249 indicates the CpG site. Because of the potential for spurious association due to chip bias, however, a randomization testing approach was used to evaluate statistical significance. For each pseudo-dataset, disease status was randomized in a two-stage process for each chip. First, disease status was randomly permuted among the subjects on that chip, and then with 50% probability, disease status was swapped between case and control for all subjects on the chip.

In each pseudo-dataset, therefore, each subject was equally likely to be assigned case or control status, which means that, 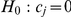 is known to be (synthetically) true. However, because the randomization is performed separately within chips, the structure of the experimental design, and therefore the chance for spurious disease association due to chip effect, is retained. The empirical null distributions resulting from this randomization approach had heavier tails than the theoretical null distribution, leading to less significant *p*-values. The randomization approach is therefore a conservative approach and properly controls type-I error in the presence of chip-to-chip variation of β-values.

To properly account for the large number of statistical tests being performed, false discovery rate (FDR) *q*-values [68] were computed for each CpG site. In our context, FDR is the proportion of detected disease associations (under a given hypothesis testing procedure) that are false, while the *q*-value is defined as the minimum FDR at which a given test can be considered significant. The *q*-value is therefore the FDR equivalent of the *p*-value and was computed using the *qvalue* package in the R statistical programming environment, under default settings [69].

### Biological Processes and Pathway Analysis

Based on the results from our association testing, a list of differentially methylated CpG sites with *p*-values less than 0.005 was generated. The CpG sites of interest were to mapped to their corresponding genes and the list of genes were then tested for potential overlaps with biological processes and pathways using the Gene Set Enrichment Analysis (GSEA) [70]. The software is designed to evaluate microarrays at the level of gene sets. Gene sets are defined as groups of genes that share common biological function, chromosomal location or regulation. The gene set database used in the analysis is established based on prior biological knowledge.

## Supporting Information

Figure S1 **Variance explained by first 20 (of 367) principal components.** The proportion of variance explained by each of the first 20 of 367 principal components, from a PCA of the 367-by-27,249 matrix of logit-transformed methylation β-values. Combined, the first 20 PCs explain 62.8% of overall variance, with no single PC among the remaining 347 explaining more than 0.34%.
(PDF)

Figure S2 **Bisulfite conversion efficiency metrics scatterplot.** Scatterplot of the red and green channels of bisulfite conversion efficiency for the 367 samples used in association testing, overlaid with a locally weighted scatterplot smoothing (LOWESS) curve.
(PDF)

Table S1 **List of differentially methylated CpG sites at P<0.005.**
(DOC)

Table S2 **Gene Set Enrichment Analysis for Biological Processes.**
(XML)

Table S3 **Gene Set Enrichment Analysis for Canonical Pathways.**
(XML)
