# Supplementary figures and images for: Maternal Genome-Wide DNA Methylation Patterns and Congenital Heart Defects
Source: PLoS One. 2011 Jan 24;6(1):e16506. doi: 10.1371/journal.pone.0016506 (PMC3031146; doi:10.1371/journal.pone.0016506)

**Variance explained by first 20 (of 367) principal components**

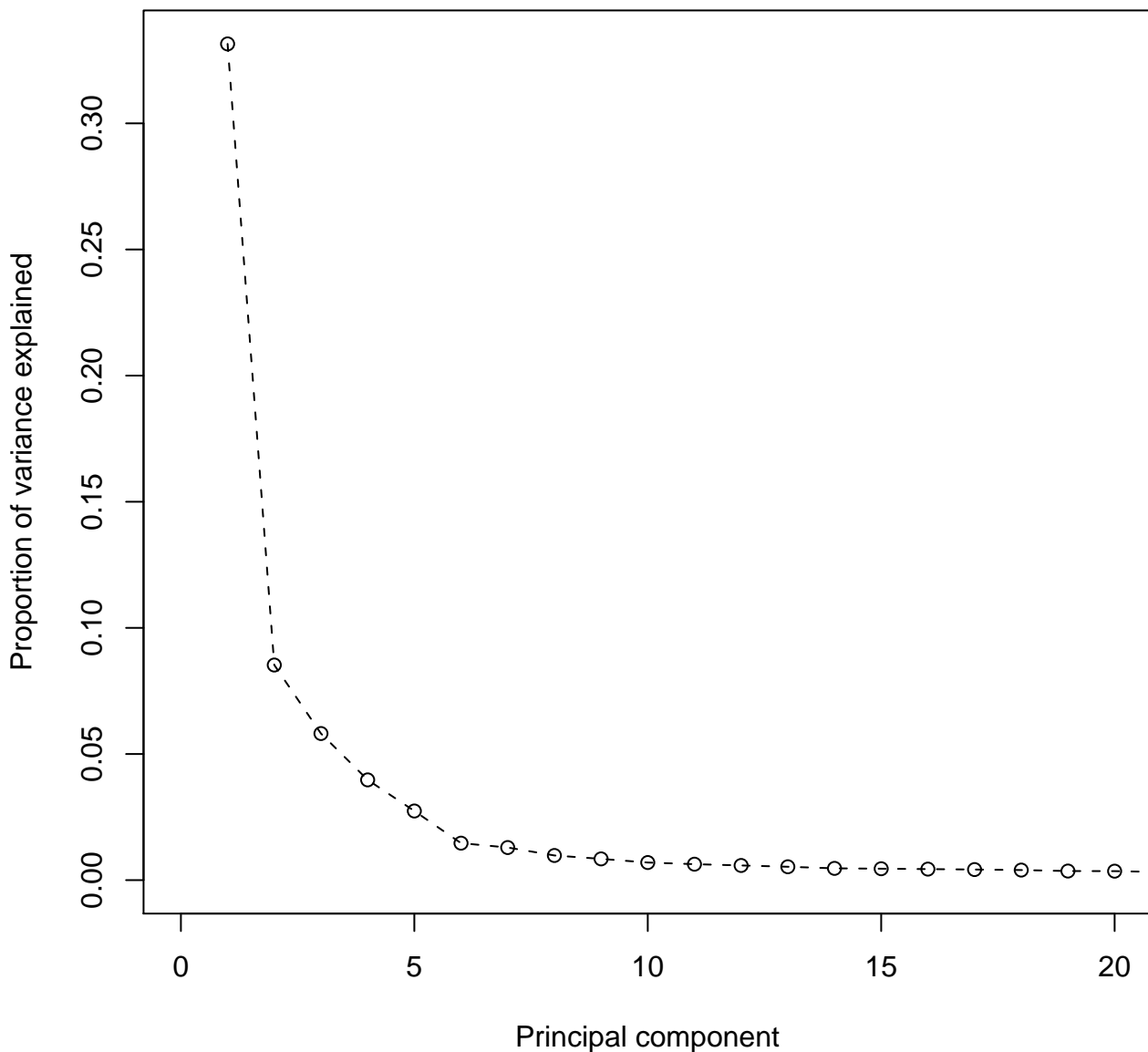

Supplement: Figure S1 — Variance explained by first 20 (of 367) principal components. The proportion of variance explained by each of the first 20 of 367 principal components, from a PCA of the 367-by-27,249 matrix of logit-transformed methylation β-values. Combined, the first 20 PCs explain 62.8% of overall variance, with no single PC among the remaining 347 explaining more than 0.34%. (PDF) [file pone.0016506.s001.pdf]

**Bisulfite conversion efficiency**  
**Red vs Green channels,  $r = 0.394$**

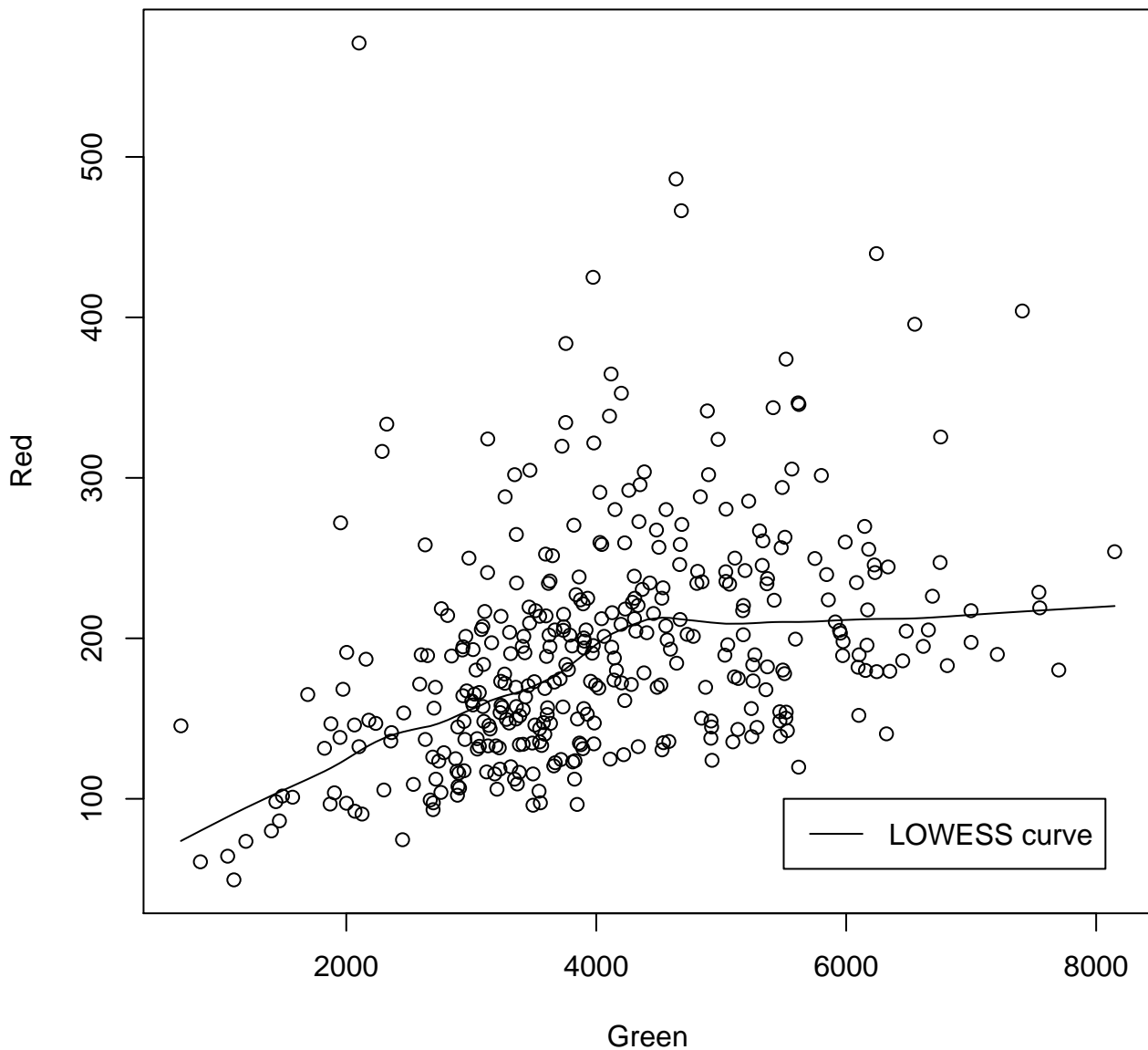

Supplement: Figure S2 — Bisulfite conversion efficiency metrics scatterplot. Scatterplot of the red and green channels of bisulfite conversion efficiency for the 367 samples used in association testing, overlaid with a locally weighted scatterplot smoothing (LOWESS) curve. (PDF) [file pone.0016506.s002.pdf]
