# Supplementary material for: Maternal Genome-Wide DNA Methylation Patterns and Congenital Heart Defects
Source: PLoS One. 2011 Jan 24;6(1):e16506. doi: 10.1371/journal.pone.0016506 (PMC3031146; doi:10.1371/journal.pone.0016506)
Supplement: Table S1 — List of differentially methylated CpG sites at P<0.005. (DOC) [file pone.0016506.s003.doc]

Table S1. List of differentially methylated CpG sites at P <0.005.

| **Target id** | **SYMBOL** | **p-value^1^** | **q-value^2^** | **Chromosome** | **CpG Island** | **Direction** |
| --- | --- | --- | --- | --- | --- | --- |
| cg02936872 | CTSL2 | 1.55E-05 | 0.133784 | 9 | Yes | + |
| cg18533466 | RREB1 | 1.63E-05 | 0.133784 | 6 | Yes | + |
| cg16933388 | BSN | 2.19E-05 | 0.133784 | 3 | Yes | + |
| cg22920873 | HSPC268 | 4.63E-05 | 0.170861 | 7 | Yes | + |
| cg11248413 | NEUROG1 | 5.46E-05 | 0.170861 | 5 | Yes | + |
| cg18440188 | MAP4K5 | 7.26E-05 | 0.170861 | 14 | Yes | + |
| cg11234457 | FTMT | 7.42E-05 | 0.170861 | 5 | Yes | - |
| cg02886284 | CPE | 8.83E-05 | 0.170861 | 4 | Yes | + |
| cg02620470 | EXT2 | 0.0001 | 0.170861 | 11 | Yes | + |
| cg23681243 | UBE3A | 0.000126 | 0.170861 | 15 | Yes | + |
| cg11695266 | DNAJB6 | 0.000141 | 0.170861 | 7 | Yes | + |
| cg21576698 | UQCRH | 0.000143 | 0.170861 | 1 | Yes | + |
| cg04135543 | ORF1-FL49 | 0.000156 | 0.170861 | 5 | Yes | + |
| cg17163751 | H2AFY2 | 0.000184 | 0.170861 | 10 | Yes | + |
| cg02558133 | PKN2 | 0.000215 | 0.170861 | 1 | Yes | + |
| cg09539408 | STRN3 | 0.000223 | 0.170861 | 14 | Yes | + |
| cg17823320 | C8orf35 | 0.000224 | 0.170861 | 8 | Yes | + |
| cg03633120 | NDUFA7 | 0.000228 | 0.170861 | 19 | Yes | - |
| cg11879480 | SLC36A4 | 0.000228 | 0.170861 | 11 | Yes | + |
| cg25513133 | C6orf72 | 0.000229 | 0.170861 | 6 | Yes | + |
| cg03976567 | C6orf199 | 0.000247 | 0.170861 | 6 | Yes | + |
| cg06540105 | CCDC74A | 0.000261 | 0.170861 | 2 | Yes | + |
| cg25396537 | TERF1 | 0.000278 | 0.170861 | 8 | Yes | + |
| cg02504465 | SNX3 | 0.000285 | 0.170861 | 6 | Yes | + |
| cg15342384 | HNRPH3 | 0.000301 | 0.170861 | 10 | Yes | + |
| cg05750321 | CGNL1 | 0.000312 | 0.170861 | 15 | Yes | - |
| cg02632166 | ITM2B | 0.000315 | 0.170861 | 13 | Yes | + |
| cg21020710 | ZNF544 | 0.000321 | 0.170861 | 19 | Yes | + |
| cg00422913 | MGC14376 | 0.000349 | 0.170861 | 17 | Yes | + |
| cg06855803 | ICAM3 | 0.000356 | 0.170861 | 19 | No | + |
| cg16612562 | RRP22 | 0.000365 | 0.170861 | 22 | Yes | + |
| cg23181138 | AGTPBP1 | 0.000366 | 0.170861 | 9 | Yes | + |
| cg15757271 | WNT5A | 0.000367 | 0.170861 | 3 | Yes | + |
| cg08932320 | GMDS | 0.000395 | 0.170861 | 6 | Yes | + |
| cg08638395 | XPR1 | 0.000403 | 0.170861 | 1 | Yes | + |
| cg15992730 | GDF3 | 0.000407 | 0.170861 | 12 | Yes | + |
| cg10319505 | RASEF | 0.000409 | 0.170861 | 9 | Yes | + |
| cg21457147 | KCTD4 | 0.00041 | 0.170861 | 13 | No | - |
| cg15318532 | RFXAP | 0.000411 | 0.170861 | 13 | Yes | + |
| cg15183083 | KCNA4 | 0.000422 | 0.170861 | 11 | Yes | + |
| cg03860890 | EGFR | 0.00043 | 0.170861 | 7 | Yes | + |
| cg23578193 | LGR6 | 0.00045 | 0.170861 | 1 | Yes | + |
| cg24155668 | MAPK13 | 0.000471 | 0.170861 | 6 | Yes | + |
| cg22992570 | PHCA | 0.000485 | 0.170861 | 11 | Yes | + |
| cg03276813 | SSFA2 | 0.000485 | 0.170861 | 2 | Yes | + |
| cg25608041 | TBC1D1 | 0.000491 | 0.170861 | 4 | Yes | + |
| cg26035366 | LRRC3B | 0.000494 | 0.170861 | 3 | Yes | + |
| cg20772904 | SHCBP1 | 0.000504 | 0.170861 | 16 | Yes | + |
| cg10485724 | PMS2 | 0.000526 | 0.170861 | 7 | Yes | + |
| cg17849956 | ZNF304 | 0.000531 | 0.170861 | 19 | Yes | + |
| cg21684385 | PEX3 | 0.000536 | 0.170861 | 6 | Yes | + |
| cg20681975 | TNRC5 | 0.000539 | 0.170861 | 6 | Yes | + |
| cg12012699 | DYM | 0.00055 | 0.170861 | 18 | Yes | + |
| cg03592161 | C17orf48 | 0.000558 | 0.170861 | 17 | Yes | + |
| cg13790571 | ABC1 | 0.000565 | 0.170861 | 17 | Yes | + |
| cg09008187 | SLC25A35 | 0.000565 | 0.170861 | 17 | Yes | + |
| cg04562739 | PCNA | 0.000565 | 0.170861 | 20 | Yes | + |
| cg17783509 | PHOX2B | 0.000583 | 0.170861 | 4 | No | - |
| cg01909921 | GFI1B | 0.000589 | 0.170861 | 9 | No | + |
| cg25395108 | FGFR1OP | 0.000593 | 0.170861 | 6 | Yes | + |
| cg19674669 | LOC112937 | 0.000602 | 0.170861 | 11 | Yes | + |
| cg08790550 | LOC112714 | 0.000607 | 0.170861 | 2 | Yes | - |
| cg17887537 | RRAGA | 0.000607 | 0.170861 | 9 | Yes | + |
| cg07054095 | ZNF549 | 0.000607 | 0.170861 | 19 | Yes | + |
| cg26426582 | TRIM60 | 0.000624 | 0.170861 | 4 | Yes | - |
| cg06320982 | FLJ10815 | 0.000629 | 0.170861 | 16 | Yes | + |
| cg06637893 | C16orf48 | 0.00064 | 0.170861 | 16 | Yes | + |
| cg07619076 | LANCL2 | 0.000661 | 0.170861 | 7 | Yes | + |
| cg07243932 | SLC30A4 | 0.000663 | 0.170861 | 15 | Yes | + |
| cg22879515 | BTG4 | 0.000666 | 0.170861 | 11 | Yes | + |
| cg05434957 | ICA1 | 0.000681 | 0.170861 | 7 | Yes | + |
| cg08332868 | RBM19 | 0.000681 | 0.170861 | 12 | Yes | + |
| cg17820459 | GPX3 | 0.000685 | 0.170861 | 5 | Yes | + |
| cg13814950 | DAPK1 | 0.000689 | 0.170861 | 9 | Yes | + |
| cg15299721 | LOC133308 | 0.00071 | 0.171959 | 4 | Yes | + |
| cg22882178 | PITX3 | 0.000712 | 0.171959 | 10 | Yes | + |
| cg13578652 | UBASH3A | 0.000731 | 0.174316 | 21 | No | - |
| cg13589108 | FAM5B | 0.000764 | 0.174398 | 1 | Yes | + |
| cg07745434 | CHST13 | 0.000789 | 0.174398 | 3 | Yes | + |
| cg00685836 | PPFIA2 | 0.000794 | 0.174398 | 12 | Yes | + |
| cg13706352 | COX10 | 0.000803 | 0.174398 | 17 | Yes | + |
| cg26864061 | SACM1L | 0.000804 | 0.174398 | 3 | Yes | + |
| cg16821394 | NDFIP1 | 0.000829 | 0.174398 | 5 | Yes | + |
| cg23257840 | ZNF264 | 0.000829 | 0.174398 | 19 | Yes | + |
| cg04053638 | SUCLG1 | 0.000833 | 0.174398 | 2 | Yes | + |
| cg08328671 | BDKRB2 | 0.000843 | 0.174398 | 14 | No | + |
| cg01428361 | RSAD1 | 0.000845 | 0.174398 | 17 | Yes | + |
| cg08369065 | GATA4 | 0.000859 | 0.174398 | 8 | Yes | + |
| cg20203395 | MGC33648 | 0.000859 | 0.174398 | 5 | Yes | + |
| cg15381769 | PTPRK | 0.000861 | 0.174398 | 6 | Yes | + |
| cg20159072 | SLC25A20 | 0.000864 | 0.174398 | 3 | Yes | + |
| cg07810967 | IL13 | 0.000913 | 0.182144 | 5 | No | - |
| cg06952310 | CSPG3 | 0.000932 | 0.182246 | 19 | No | - |
| cg22980079 | C4orf8 | 0.000944 | 0.182246 | 4 | No | - |
| cg02612026 | CASP9 | 0.000966 | 0.182246 | 1 | Yes | + |
| cg18825999 | PNRC2 | 0.000972 | 0.182246 | 1 | Yes | + |
| cg16413535 | TKTL2 | 0.000975 | 0.182246 | 4 | Yes | - |
| cg03391040 | ZNF420 | 0.000992 | 0.182246 | 19 | Yes | + |
| cg06746101 | TMED3 | 0.001015 | 0.182246 | 15 | Yes | + |
| cg13289561 | DKFZP586D0919 | 0.001032 | 0.182246 | 12 | Yes | + |
| cg03958344 | CNP | 0.001059 | 0.182246 | 17 | Yes | + |
| cg27443050 | DLG7 | 0.00107 | 0.182246 | 14 | Yes | - |
| cg06953768 | FLJ37562 | 0.001072 | 0.182246 | 5 | Yes | + |
| cg19220754 | TNPO1 | 0.001076 | 0.182246 | 5 | Yes | + |
| cg17316750 | FAM82C | 0.001109 | 0.182246 | 15 | Yes | + |
| cg12619162 | FXYD4 | 0.001119 | 0.182246 | 10 | No | - |
| cg15092544 | RAP1B | 0.001132 | 0.182246 | 12 | Yes | + |
| cg04623168 | C12orf30 | 0.001144 | 0.182246 | 12 | Yes | + |
| cg11976048 | MGC4172 | 0.001144 | 0.182246 | 17 | Yes | + |
| cg02699167 | FBXL2 | 0.00115 | 0.182246 | 3 | Yes | + |
| cg02975142 | FLCN | 0.001174 | 0.182246 | 17 | Yes | + |
| cg20263942 | GOLPH2 | 0.001188 | 0.182246 | 9 | Yes | + |
| cg02342494 | MGC50559 | 0.001217 | 0.182246 | 12 | Yes | + |
| cg02888247 | C6orf62 | 0.001247 | 0.182246 | 6 | No | - |
| cg20326867 | STIM2 | 0.001247 | 0.182246 | 4 | Yes | + |
| cg15002204 | C6orf139 | 0.001255 | 0.182246 | 6 | No | + |
| cg06953304 | NKTR | 0.001258 | 0.182246 | 3 | Yes | + |
| cg09068528 | ACADL | 0.001292 | 0.182246 | 2 | Yes | + |
| cg26762198 | C16orf9 | 0.001304 | 0.182246 | 16 | Yes | + |
| cg11916186 | THRAP3 | 0.001313 | 0.182246 | 1 | Yes | + |
| cg05978187 | ERH | 0.001318 | 0.182246 | 14 | Yes | + |
| cg16434306 | FLJ14054 | 0.001337 | 0.182246 | 5 | No | - |
| cg15785580 | BCKDK | 0.001362 | 0.182246 | 16 | Yes | + |
| cg13877465 | IGF2BP1 | 0.001362 | 0.182246 | 17 | Yes | + |
| cg20918903 | JMJD1A | 0.001362 | 0.182246 | 2 | Yes | + |
| cg06302803 | MATR3 | 0.001363 | 0.182246 | 5 | Yes | + |
| cg22984616 | BAG2 | 0.001364 | 0.182246 | 6 | Yes | + |
| cg20469837 | GALNT5 | 0.001425 | 0.182246 | 2 | No | + |
| cg15600835 | raptor | 0.001445 | 0.182246 | 17 | Yes | + |
| cg13446852 | LEPR | 0.00145 | 0.182246 | 1 | Yes | + |
| cg23481221 | TOMM34 | 0.001453 | 0.182246 | 20 | Yes | + |
| cg11332950 | AHSA2 | 0.001473 | 0.182246 | 2 | Yes | + |
| cg26651830 | BCAP29 | 0.001473 | 0.182246 | 7 | Yes | + |
| cg03713346 | CPNE2 | 0.001473 | 0.182246 | 16 | Yes | + |
| cg19552482 | ZNF691 | 0.001477 | 0.182246 | 1 | Yes | + |
| cg09006267 | ACAT1 | 0.001487 | 0.182246 | 11 | Yes | + |
| cg17597195 | TMEM126B | 0.001487 | 0.182246 | 11 | Yes | + |
| cg07749808 | SYT17 | 0.001494 | 0.182246 | 16 | Yes | + |
| cg09238677 | C3AR1 | 0.001496 | 0.182246 | 12 | No | + |
| cg26043391 | FBXO28 | 0.001506 | 0.182246 | 1 | Yes | + |
| cg26709720 | B3GALT5 | 0.001516 | 0.182246 | 21 | No | - |
| cg12024906 | HKR1 | 0.001528 | 0.182246 | 19 | Yes | + |
| cg24989962 | PTGDR | 0.001541 | 0.182246 | 14 | Yes | + |
| cg00469635 | PPME1 | 0.001558 | 0.182246 | 11 | Yes | + |
| cg08788717 | STK33 | 0.001558 | 0.182246 | 11 | Yes | + |
| cg13022174 | SS18 | 0.001569 | 0.182246 | 18 | Yes | - |
| cg03403539 | NUPL2 | 0.001579 | 0.182246 | 7 | Yes | + |
| cg06921282 | LRRC56 | 0.001595 | 0.182246 | 11 | Yes | + |
| cg25077654 | DSCR2 | 0.001621 | 0.182246 | 21 | Yes | + |
| cg22631642 | POLR2J2 | 0.001621 | 0.182246 | 7 | Yes | + |
| cg14443380 | SEMA7A | 0.001621 | 0.182246 | 15 | Yes | + |
| cg15240064 | ZNF12 | 0.001621 | 0.182246 | 7 | Yes | + |
| cg11653500 | SPRY2 | 0.001624 | 0.182246 | 13 | Yes | + |
| cg07559730 | ZNF702 | 0.00163 | 0.182246 | 19 | Yes | + |
| cg07699440 | FAH | 0.001652 | 0.182246 | 15 | Yes | + |
| cg27534796 | AIG1 | 0.001684 | 0.182246 | 6 | Yes | + |
| cg12683929 | BLOC1S1 | 0.001686 | 0.182246 | 12 | No | + |
| cg15103675 | UAP1 | 0.0017 | 0.182246 | 1 | Yes | + |
| cg01171588 | USP38 | 0.001744 | 0.182246 | 4 | Yes | + |
| cg20028291 | BCAS4 | 0.001791 | 0.182246 | 20 | Yes | + |
| cg02668581 | DSU | 0.001791 | 0.182246 | 2 | Yes | + |
| cg15945417 | LLGL1 | 0.001791 | 0.182246 | 17 | Yes | + |
| cg04725234 | TMEM38B | 0.001815 | 0.182246 | 9 | Yes | + |
| cg13060405 | MYO18A | 0.001817 | 0.182246 | 17 | Yes | + |
| cg10891157 | CRTAP | 0.001819 | 0.182246 | 3 | Yes | + |
| cg07294870 | PIGT | 0.001822 | 0.182246 | 20 | Yes | + |
| cg25591867 | CKAP4 | 0.001846 | 0.182246 | 12 | Yes | + |
| cg26301689 | PAQR9 | 0.001857 | 0.182246 | 3 | Yes | - |
| cg06600412 | MLF1IP | 0.001874 | 0.182246 | 4 | Yes | + |
| cg10318443 | RNMTL1 | 0.001892 | 0.182246 | 17 | Yes | + |
| cg20098887 | RAB32 | 0.001903 | 0.182246 | 6 | Yes | + |
| cg04941721 | EREG | 0.001927 | 0.182246 | 4 | Yes | + |
| cg11159727 | MTERF | 0.001951 | 0.182246 | 7 | No | + |
| cg08430598 | CST1 | 0.001981 | 0.182246 | 20 | No | - |
| cg15344028 | ICOS | 0.001983 | 0.182246 | 2 | No | - |
| cg23361608 | DSCR5 | 0.001984 | 0.182246 | 21 | Yes | + |
| cg10211877 | PDRG1 | 0.001992 | 0.182246 | 20 | Yes | + |
| cg02326931 | ALG5 | 0.002005 | 0.182246 | 13 | Yes | + |
| cg26162695 | ELAC2 | 0.002005 | 0.182246 | 17 | Yes | + |
| cg08286169 | PEX3 | 0.002005 | 0.182246 | 6 | Yes | + |
| cg06786424 | PRPF4B | 0.002017 | 0.182246 | 6 | Yes | + |
| cg16878021 |  | 0.00202 | 0.182246 | 14 | No | - |
| cg23700778 | KIAA0895 | 0.002024 | 0.182246 | 7 | Yes | + |
| cg23346960 | ZFP36 | 0.002024 | 0.182246 | 19 | Yes | + |
| cg18801292 | SUPT16H | 0.002074 | 0.182246 | 14 | Yes | + |
| cg04633384 | C18orf1 | 0.0021 | 0.182246 | 18 | Yes | + |
| cg11648289 | EN2 | 0.002129 | 0.182246 | 7 | Yes | + |
| cg12884406 | COL11A1 | 0.002139 | 0.182246 | 1 | Yes | + |
| cg11781389 | LOC89944 | 0.002139 | 0.182246 | 11 | Yes | + |
| cg25860314 | SEC24D | 0.002154 | 0.182246 | 4 | Yes | + |
| cg19439399 | ELOVL4 | 0.002161 | 0.182246 | 6 | Yes | + |
| cg10605520 | HRH3 | 0.002161 | 0.182246 | 20 | Yes | + |
| cg14732540 | BRDT | 0.002166 | 0.182246 | 1 | Yes | - |
| cg00024812 | CPSF3 | 0.002172 | 0.182246 | 2 | Yes | + |
| cg26608032 | MKL1 | 0.002175 | 0.182246 | 22 | Yes | + |
| cg22858728 | MPP1 | 0.002192 | 0.182246 |  | Yes | - |
| cg22830895 | CRYGN | 0.002216 | 0.182246 | 7 | No | + |
| cg02863842 | PVRL2 | 0.002227 | 0.182246 | 19 | Yes | + |
| cg21197973 | PH-4 | 0.002232 | 0.182246 | 3 | Yes | + |
| cg22864416 | CCNJ | 0.002233 | 0.182246 | 10 | Yes | + |
| cg24183484 | GGN | 0.002243 | 0.182246 | 19 | Yes | + |
| cg17506742 | IL10RB | 0.002243 | 0.182246 | 21 | Yes | + |
| cg12839593 | SIX1 | 0.002243 | 0.182246 | 14 | Yes | + |
| cg22813220 | C1orf83 | 0.002278 | 0.182246 | 1 | Yes | + |
| cg21984310 | C9orf127 | 0.002278 | 0.182246 | 9 | Yes | + |
| cg00210842 | APAF1 | 0.002281 | 0.182246 | 12 | Yes | + |
| cg25922239 | LEMD2 | 0.002281 | 0.182246 | 6 | Yes | + |
| cg21491028 | PSMD3 | 0.002341 | 0.182246 | 17 | Yes | + |
| cg23489497 | ATPBD1B | 0.002353 | 0.182246 | 1 | Yes | + |
| cg16417937 | NUDC | 0.002353 | 0.182246 | 1 | Yes | + |
| cg04148458 | FAIM | 0.002359 | 0.182246 | 3 | Yes | + |
| cg15819171 | FLJ23588 | 0.002359 | 0.182246 | 22 | Yes | + |
| cg09874127 | UBE1L | 0.002395 | 0.182246 | 3 | No | + |
| cg00688421 | RAP1GDS1 | 0.002397 | 0.182246 | 4 | Yes | - |
| cg15119375 | MGC3020 | 0.002398 | 0.182246 | 16 | Yes | + |
| cg01090445 | TRAIP | 0.002398 | 0.182246 | 3 | Yes | + |
| cg20991095 | USP49 | 0.002398 | 0.182246 | 6 | Yes | + |
| cg16025584 | C9orf142 | 0.002407 | 0.182246 | 9 | Yes | + |
| cg03742214 | CRY1 | 0.002432 | 0.182246 | 12 | Yes | + |
| cg07570421 | PTER | 0.002448 | 0.182246 | 10 | Yes | + |
| cg22289360 | GCNT1 | 0.002457 | 0.182246 | 9 | Yes | + |
| cg18383160 | C14orf24 | 0.002466 | 0.182246 | 14 | Yes | + |
| cg00528052 | JARID1B | 0.00248 | 0.182246 | 1 | Yes | + |
| cg09370442 | CHP | 0.002488 | 0.182246 | 15 | Yes | + |
| cg15321195 | AGPAT3 | 0.002492 | 0.182246 | 21 | Yes | + |
| cg15307268 | GPR176 | 0.002492 | 0.182246 | 15 | Yes | + |
| cg08567916 | SPRYD3 | 0.002492 | 0.182246 | 12 | Yes | + |
| cg26820922 | YIPF4 | 0.002492 | 0.182246 | 2 | Yes | + |
| cg12465398 | PPIL3 | 0.002497 | 0.182246 | 2 | Yes | + |
| cg08367703 | SDCCAG3 | 0.002497 | 0.182246 | 9 | Yes | + |
| cg07847428 | 2-Mar | 0.002513 | 0.182246 | 19 | Yes | + |
| cg05156613 | ACTL7B | 0.002545 | 0.182246 | 9 | Yes | - |
| cg02100629 | AMID | 0.002574 | 0.182246 | 10 | Yes | + |
| cg07274506 | NOL9 | 0.002574 | 0.182246 | 1 | Yes | + |
| cg09768944 | PDCL | 0.002601 | 0.182246 | 9 | Yes | + |
| cg07708788 | SLCO2A1 | 0.002608 | 0.182246 | 3 | Yes | + |
| cg02255004 | GDEP | 0.002613 | 0.182246 | 4 | No | + |
| cg10830758 | C10orf4 | 0.002614 | 0.182246 | 10 | Yes | + |
| cg03062665 | CALM1 | 0.002614 | 0.182246 | 14 | Yes | + |
| cg14309281 | MAP3K3 | 0.002614 | 0.182246 | 17 | Yes | + |
| cg17261830 | NEK9 | 0.002614 | 0.182246 | 14 | Yes | + |
| cg16829450 | NTN4 | 0.002614 | 0.182246 | 12 | Yes | + |
| cg15247144 | PYCARD | 0.002614 | 0.182246 | 16 | Yes | + |
| cg10377451 | RAB33B | 0.002614 | 0.182246 | 4 | Yes | + |
| cg27550442 | ZNHIT1 | 0.002614 | 0.182246 | 7 | Yes | + |
| cg07874155 | CTNNA1 | 0.002618 | 0.182246 | 5 | Yes | + |
| cg00836605 | JAZF1 | 0.002618 | 0.182246 | 7 | Yes | + |
| cg04970117 | SLC6A20 | 0.002618 | 0.182246 | 3 | Yes | + |
| cg19352038 | PAX3 | 0.002633 | 0.182246 | 2 | Yes | + |
| cg26701198 | COG6 | 0.002635 | 0.182246 | 13 | Yes | + |
| cg20959523 | FIGN | 0.002635 | 0.182246 | 2 | Yes | + |
| cg15492104 | C14orf44 | 0.00264 | 0.182246 | 14 | Yes | + |
| cg18050213 | FAM14A | 0.002642 | 0.182246 | 14 | Yes | + |
| cg04185893 | PTBP2 | 0.002668 | 0.182246 | 1 | Yes | + |
| cg00292662 | LGALS1 | 0.002674 | 0.182246 | 22 | No | + |
| cg22201387 | ENTPD5 | 0.002679 | 0.182246 | 14 | Yes | + |
| cg25985488 | GALNTL4 | 0.002679 | 0.182246 | 11 | Yes | + |
| cg07314549 | MGC16943 | 0.002679 | 0.182246 | 16 | Yes | + |
| cg27555479 | DYNLRB2 | 0.002716 | 0.182246 | 16 | Yes | + |
| cg04532229 | SLC9A2 | 0.002725 | 0.182246 | 2 | Yes | + |
| cg14681055 | PITX3 | 0.002776 | 0.182246 | 10 | Yes | + |
| cg13730534 | NFYC | 0.002797 | 0.182246 | 1 | Yes | + |
| cg10488637 | ZNF180 | 0.002833 | 0.182246 | 19 | Yes | + |
| cg01352108 | KCNK4 | 0.00288 | 0.182246 | 11 | Yes | + |
| cg27501458 | SULT4A1 | 0.002894 | 0.182246 | 22 | Yes | + |
| cg24315860 | SRPK2 | 0.002909 | 0.182246 | 7 | Yes | + |
| cg07572435 | LY6D | 0.002946 | 0.182246 | 8 | No | - |
| cg17723549 | CCDC55 | 0.002954 | 0.182246 | 17 | Yes | - |
| cg22884082 | GJB7 | 0.002967 | 0.182246 | 6 | Yes | - |
| cg13332088 | C14orf80 | 0.002967 | 0.182246 | 14 | Yes | + |
| cg08113913 | CCDC74B | 0.002967 | 0.182246 | 2 | Yes | + |
| cg21682826 | HERPUD1 | 0.002967 | 0.182246 | 16 | Yes | + |
| cg05181565 | SSU72 | 0.002967 | 0.182246 | 1 | Yes | + |
| cg17471928 | STAC2 | 0.002967 | 0.182246 | 17 | Yes | + |
| cg10166697 | SLC25A13 | 0.002996 | 0.182246 | 7 | Yes | + |
| cg13788301 | TOM1 | 0.00301 | 0.182246 | 22 | Yes | + |
| cg18278261 | FUS | 0.003014 | 0.182246 | 16 | Yes | + |
| cg01725199 | GALNT12 | 0.003014 | 0.182246 | 9 | Yes | + |
| cg27393372 | SCRN3 | 0.003014 | 0.182246 | 2 | Yes | + |
| cg05593479 | TIGD1 | 0.003014 | 0.182246 | 2 | Yes | + |
| cg18759346 | COMMD2 | 0.00303 | 0.182246 | 3 | Yes | + |
| cg08646988 | GFPT1 | 0.00303 | 0.182246 | 2 | Yes | + |
| cg05421555 | AKAP13 | 0.003115 | 0.182246 | 15 | Yes | + |
| cg07477160 | NDRG1 | 0.003155 | 0.182246 | 8 | Yes | + |
| cg01803810 | TSEN54 | 0.003155 | 0.182246 | 17 | Yes | + |
| cg17731079 | CDC42EP3 | 0.003157 | 0.182246 | 2 | Yes | + |
| cg10484958 | PCDH8 | 0.003208 | 0.182246 | 13 | Yes | + |
| cg27367952 | MGC50811 | 0.00326 | 0.182246 | 2 | No | + |
| cg07567107 | ABC1 | 0.003276 | 0.182246 | 17 | Yes | + |
| cg03700462 | HOXA1 | 0.003295 | 0.182246 | 7 | Yes | + |
| cg04344347 | ADI1 | 0.003306 | 0.182246 | 2 | Yes | + |
| cg12796229 | C18orf43 | 0.003306 | 0.182246 | 18 | Yes | + |
| cg02271621 | DUSP8 | 0.003306 | 0.182246 | 11 | Yes | + |
| cg00696900 | MAT2A | 0.003306 | 0.182246 | 2 | Yes | + |
| cg20947775 | SCD5 | 0.003306 | 0.182246 | 4 | Yes | + |
| cg00368415 | SNX27 | 0.003306 | 0.182246 | 1 | Yes | + |
| cg14838256 | SRD5A2L | 0.003306 | 0.182246 | 4 | Yes | + |
| cg13983870 | TSPAN12 | 0.003306 | 0.182246 | 7 | Yes | + |
| cg01803238 | BCL2 | 0.003325 | 0.182246 | 18 | Yes | + |
| cg09896445 | GPC5 | 0.003325 | 0.182246 | 13 | Yes | + |
| cg11913104 | RAD23A | 0.003331 | 0.182246 | 19 | Yes | + |
| cg00471562 | WDR37 | 0.003331 | 0.182246 | 10 | Yes | + |
| cg14623805 | CCAR1 | 0.003404 | 0.182246 | 10 | Yes | + |
| cg18070061 | DUSP4 | 0.003404 | 0.182246 | 8 | Yes | + |
| cg04195527 | INSIG2 | 0.003434 | 0.182246 | 2 | Yes | + |
| cg08473533 | MGC24381 | 0.003454 | 0.182246 | 16 | Yes | + |
| cg24744425 | RABIF | 0.003454 | 0.182246 | 1 | Yes | + |
| cg20477318 | TIMM23 | 0.00349 | 0.182246 | 10 | Yes | + |
| cg23441208 | BCAT2 | 0.003503 | 0.182246 | 19 | Yes | + |
| cg25304860 | C14orf166 | 0.003503 | 0.182246 | 14 | Yes | + |
| cg18085206 | CRK | 0.003503 | 0.182246 | 17 | Yes | + |
| cg09429111 | PTPDC1 | 0.003503 | 0.182246 | 9 | Yes | + |
| cg23564033 | THOC3 | 0.003559 | 0.182246 | 5 | Yes | + |
| cg22740783 | CGREF1 | 0.003559 | 0.182246 | 2 | No | + |
| cg26381263 | PPP2R2D | 0.003581 | 0.182246 | 10 | No | - |
| cg10862848 | GNMT | 0.003584 | 0.182246 | 6 | Yes | + |
| cg03588357 | GPR68 | 0.003594 | 0.182246 | 14 | Yes | + |
| cg15905979 | ST18 | 0.003604 | 0.182246 | 8 | Yes | - |
| cg00848728 | DAB1 | 0.00363 | 0.182246 | 1 | Yes | + |
| cg22024876 | KBTBD3 | 0.00363 | 0.182246 | 11 | Yes | + |
| cg16964535 | DNAJC5G | 0.003656 | 0.182246 | 2 | Yes | - |
| cg20884697 | MANEA | 0.00366 | 0.182246 | 6 | Yes | + |
| cg22827640 | HPCAL4 | 0.00367 | 0.182246 | 1 | Yes | + |
| cg04206644 | DDX26 | 0.003683 | 0.182246 | 13 | Yes | + |
| cg25640176 | MGC11335 | 0.003697 | 0.182246 | 16 | Yes | + |
| cg00539716 | HTLF | 0.003724 | 0.182246 | 2 | Yes | + |
| cg06110728 | NELL2 | 0.003738 | 0.182246 | 12 | Yes | + |
| cg06376129 | RIPK5 | 0.003774 | 0.182246 | 1 | Yes | + |
| cg23029491 | UBAP2 | 0.003777 | 0.182246 | 9 | Yes | + |
| cg03672021 | FLNA | 0.003788 | 0.182246 |  | Yes | - |
| cg00012199 | RNASE4 | 0.003788 | 0.182246 | 14 | Yes | + |
| cg09503974 | RARRES1 | 0.003831 | 0.182246 | 3 | Yes | + |
| cg07519011 | TBC1D4 | 0.003857 | 0.182246 | 13 | Yes | + |
| cg08724517 | FLJ21159 | 0.003865 | 0.182246 | 4 | Yes | + |
| cg03812679 | CSF3 | 0.003887 | 0.182246 | 17 | Yes | + |
| cg15520279 | HOXD8 | 0.003887 | 0.182246 | 2 | Yes | + |
| cg25782563 | MED9 | 0.003887 | 0.182246 | 17 | Yes | + |
| cg18357098 | C3orf19 | 0.003899 | 0.182246 | 3 | Yes | + |
| cg00891278 | CCDC37 | 0.003899 | 0.182246 | 3 | Yes | + |
| cg20478514 | SCCPDH | 0.003925 | 0.182246 | 1 | Yes | + |
| cg18140857 | RDHE2 | 0.003933 | 0.182246 | 8 | Yes | - |
| cg18506672 | SNURF | 0.003953 | 0.182246 | 15 | Yes | - |
| cg16482226 | CNOT2 | 0.003953 | 0.182246 | 12 | Yes | + |
| cg15948326 | C11orf45 | 0.003988 | 0.182246 | 11 | Yes | + |
| cg23654549 | CD24 | 0.003992 | 0.182246 |  | Yes | + |
| cg11059483 | ESR2 | 0.003992 | 0.182246 | 14 | Yes | + |
| cg07159490 | NXPH4 | 0.003992 | 0.182246 | 12 | Yes | + |
| cg16148403 | SLA/LP | 0.003992 | 0.182246 | 4 | Yes | + |
| cg21235838 | SORBS1 | 0.003992 | 0.182246 | 10 | Yes | + |
| cg21309049 | STK17A | 0.003992 | 0.182246 | 7 | Yes | + |
| cg02738763 | TARSL2 | 0.003992 | 0.182246 | 15 | Yes | + |
| cg23459997 | JAGN1 | 0.004004 | 0.182246 | 3 | Yes | + |
| cg13904493 | KIAA1804 | 0.004004 | 0.182246 | 1 | Yes | + |
| cg25465406 | GUCY2D | 0.004024 | 0.182246 | 17 | Yes | + |
| cg11326613 | MGC31967 | 0.004024 | 0.182246 | 9 | Yes | + |
| cg05764376 | THAP10 | 0.004057 | 0.182246 | 15 | Yes | + |
| cg23706211 | CELSR2 | 0.004082 | 0.182246 | 1 | Yes | + |
| cg13384396 | ADCY5 | 0.004094 | 0.182246 | 3 | Yes | + |
| cg24654547 | DUS2L | 0.004132 | 0.182246 | 16 | Yes | - |
| cg18342279 | ZAR1 | 0.004132 | 0.182246 | 4 | Yes | + |
| cg02435593 | CHMP4B | 0.004141 | 0.182246 | 20 | Yes | - |
| cg11179184 | C8orf76 | 0.004154 | 0.182246 | 8 | Yes | - |
| cg16046465 | FRMD5 | 0.004154 | 0.182246 | 15 | Yes | + |
| cg22030890 | RAB32 | 0.004171 | 0.182246 | 6 | Yes | + |
| cg10098541 | PDIA5 | 0.00418 | 0.182246 | 3 | Yes | + |
| cg07017706 | K6IRS3 | 0.004197 | 0.182246 | 12 | No | - |
| cg04763192 | C1orf88 | 0.004233 | 0.182246 | 1 | Yes | + |
| cg20315136 | RGS2 | 0.004264 | 0.182246 | 1 | Yes | + |
| cg24642468 | MGC33367 | 0.004269 | 0.182246 | 16 | No | + |
| cg03139377 | SLC6A12 | 0.004324 | 0.182246 | 12 | No | + |
| cg21321885 | NDE1 | 0.004329 | 0.182246 | 16 | Yes | + |
| cg21546671 | HOXB4 | 0.004338 | 0.182246 | 17 | Yes | + |
| cg10970409 | VIPR1 | 0.004348 | 0.182246 | 3 | Yes | - |
| cg25336198 | INS | 0.004391 | 0.182246 | 11 | No | - |
| cg09420988 | MRPS30 | 0.004435 | 0.182246 | 5 | Yes | + |
| cg23123694 | SLC38A2 | 0.004449 | 0.182246 | 12 | Yes | + |
| cg14281165 | PIGO | 0.004454 | 0.182246 | 9 | Yes | + |
| cg24237439 | FLJ25422 | 0.004459 | 0.182246 | 5 | No | + |
| cg09787254 | FA2H | 0.004464 | 0.182246 | 16 | Yes | + |
| cg16085042 | HSP90B1 | 0.004464 | 0.182246 | 12 | Yes | + |
| cg01598046 | TRAIP | 0.004464 | 0.182246 | 3 | Yes | + |
| cg04876451 | PET112L | 0.004505 | 0.182246 | 4 | Yes | + |
| cg14473016 | RASD2 | 0.004535 | 0.182246 | 22 | Yes | + |
| cg18338311 | LOC124842 | 0.00454 | 0.182246 | 17 | Yes | + |
| cg08434234 | DGKI | 0.004546 | 0.182246 | 7 | Yes | + |
| cg18654873 | MAP3K7 | 0.004546 | 0.182246 | 6 | Yes | + |
| cg19310430 | C11orf45 | 0.004546 | 0.182246 | 11 | No | + |
| cg19196684 | SLC1A7 | 0.004556 | 0.182246 | 1 | No | - |
| cg19988449 | BNC1 | 0.004577 | 0.182246 | 15 | Yes | + |
| cg11763112 | C6orf84 | 0.004577 | 0.182246 | 6 | Yes | + |
| cg10126874 | CRY1 | 0.004577 | 0.182246 | 12 | Yes | + |
| cg08250444 | EFNA1 | 0.004577 | 0.182246 | 1 | Yes | + |
| cg13031679 | FLJ10560 | 0.004577 | 0.182246 | 3 | Yes | + |
| cg22996555 | GCS1 | 0.004577 | 0.182246 | 2 | Yes | + |
| cg24070847 | HIST2H2BE | 0.004577 | 0.182246 | 1 | Yes | + |
| cg12917695 | HSPC268 | 0.004577 | 0.182246 | 7 | Yes | + |
| cg16228804 | PKN2 | 0.004577 | 0.182246 | 1 | Yes | + |
| cg21289015 | USH1C | 0.004582 | 0.182246 | 11 | Yes | + |
| cg15945769 | HHAT | 0.004587 | 0.182246 | 1 | Yes | + |
| cg11462865 | KRT19 | 0.004651 | 0.182246 | 17 | Yes | + |
| cg16641915 | CAPN5 | 0.004657 | 0.182246 | 11 | Yes | + |
| cg00581156 | HSA9761 | 0.004678 | 0.182246 | 5 | Yes | + |
| cg10681725 | RPS23 | 0.004678 | 0.182246 | 5 | Yes | + |
| cg25638984 | TMTC2 | 0.004678 | 0.182246 | 12 | Yes | + |
| cg23258063 | SCYL3 | 0.004711 | 0.182246 | 1 | Yes | + |
| cg10948777 | DNM2 | 0.004734 | 0.182246 | 19 | Yes | + |
| cg04894993 | KIAA0265 | 0.004734 | 0.182246 | 7 | Yes | + |
| cg19110684 | HAVCR2 | 0.004734 | 0.182246 | 5 | No | + |
| cg07070934 | GJA4 | 0.004739 | 0.182246 | 1 | Yes | + |
| cg12441964 | FLJ35894 | 0.004773 | 0.182246 | 16 | Yes | - |
| cg04125208 | CD44 | 0.00479 | 0.182246 | 11 | Yes | + |
| cg17341358 | MRPL1 | 0.004808 | 0.182246 | 4 | Yes | + |
| cg04963951 | FTMT | 0.004819 | 0.182246 | 5 | Yes | - |
| cg04785461 | DCLRE1C | 0.004819 | 0.182246 | 10 | Yes | + |
| cg22198623 | MT1G | 0.004819 | 0.182246 | 16 | Yes | + |
| cg09633588 | C1orf174 | 0.004843 | 0.182246 | 1 | Yes | + |
| cg26916927 | GLO1 | 0.004843 | 0.182246 | 6 | Yes | + |
| cg01137737 | MIR16 | 0.004843 | 0.182246 | 16 | Yes | + |
| cg01802635 | TXNDC5 | 0.004843 | 0.182246 | 6 | Yes | + |
| cg08817171 | KIF23 | 0.004854 | 0.182246 | 15 | Yes | + |
| cg15127806 | MRPL42 | 0.004854 | 0.182246 | 12 | Yes | + |
| cg02946754 | NHLRC2 | 0.00486 | 0.182246 | 10 | Yes | + |
| cg23909633 | IL24 | 0.004932 | 0.182246 | 1 | No | - |
| cg18127191 | CDC7 | 0.004932 | 0.182246 | 1 | Yes | + |
| cg01680823 | TCAP | 0.004969 | 0.182246 | 17 | No | - |

^1^ p-values were calculated by multiple linear regression and randomization testing (see Materials and Methods).

^2^ q-values were calculated using the *qvalue* package in the R statistical programming environment under default settings (see Materials and Methods).
